# A Novel Strategy for Understanding What Patients Value Most in Informed Consent Before Surgery

**DOI:** 10.3390/healthcare13050534

**Published:** 2025-02-28

**Authors:** Gillie Gabay, Attila Gere, Glenn Zemel, Howard Moskowitz

**Affiliations:** 1 School of Sciences, Multi-Disciplinary Studies, Achva Academic College, Arugot 7980400, Israel; 2 Institute of Food Science and Technology, Hungarian University of Agriculture and Life Sciences, 1118 Budapest, Hungary; gere.attila@uni-mate.hu; 3 Independent Researcher, Naperville, IL 60540, USA; 4 Tactical Data Group, Stafford, VA 22554, USA; mjhrm@gmail.com

**Keywords:** anxiety, communication, experimental design, patient perspective, pre-operative informed consent, patient voice

## Abstract

**Background/Objectives**: To map and analyze patient expectations regarding communication in IC and identify communication that both heightens anxiety in the IC process and reduces anxiety in the IC process before surgery. **Methods**: Ethics approval was granted. A power analysis indicated a required sample of 90 patients. A conjoint-based experimental design was performed, post-discharge, overcoming typical biases of surveys. **Results**: The sample comprised 104 patients who underwent surgery in the last year. Three verbal communication messages were perceived as significantly decreasing pre-operative anxiety for the total sample. Mathematical clustering yielded three distinct mindsets. Post hoc ANOVA indices indicated that the mindsets were significantly different. Patients belonging to each mindset differed from patients belonging to other mindsets in their expectations from the dialogue with surgeons to mitigate their anxiety. Mindset 1 (70% of the sample) comprised patients who expected information that was tailored to their specific situation. To feel safer, they needed to know that nothing unexpected would happen. Mindset 2 (13%) comprised patients who expected providers to talk with them about benefits and risks at the clinic, not at the hospital, and have a dialogue with them. Mindset 3 (17%) comprised patients who perceived a lack of information regarding the purpose of signing the informed consent and lack of sufficient time to thoroughly read the form or signing the form minutes before the procedure as elements that would heighten their anxiety. **Conclusions**: Three verbal communication messages in the IC dialogue were thought to decrease pre-operative anxiety for all patients, as follows. “I want to make sure you read and understand the consent form entirely” “Everything is provided in clear and simple terms”. The surgeon says, “Let’s go over the entire form”.

## 1. Introduction

Informed consent (IC) is rooted in the principles of respecting patient autonomy and recognizing their right to define personal goals and limitations when choosing a medical procedure. The autonomy to accept or decline specific procedure underscores why IC is often described as “informed choice”, serving as a cornerstone of medical ethics [1,2,3]. Legally and ethically, obtaining IC is a long-established requirement before performing any invasive procedure [4]. Patients must provide consent because, while procedures aim to enhance their health, they also carry potential risks of adverse outcomes. For IC to be considered valid, it must be given voluntarily by a patient who has the capacity to consent. This entails the patient: comprehending the information presented, retaining it long enough to make an informed decision, evaluating the information as part of their decision-making process, and clearly communicating their choice [1,5]. Achieving IC involves a collaborative dialogue between physician and patient, ensuring the patient fully understands their health condition, the medical facts, the proposed procedure and its details, potential risks and benefits, alternatives, consequences of non-treatment, as well as the diagnosis, prognosis, and treatment progress. Only after this comprehensive discussion can the patient decide to proceed with or decline the procedure [4,6]. The signed IC form is evidence of consent, but it is often an oversimplification, since there is a risk of acquiescence by a patient who may not be fully informed [4]. Thus, IC is much more than a signature [6,7]. Physicians are responsible by law to act upon principles of medical ethics and provide patients with complete and accurate information before the procedure to enable their decision-making.

Physicians follow three key criteria to ensure adequate informed consent (IC): capacity, autonomy, and disclosure [8]. *Capacity* refers to the patient’s mental ability to make informed decisions, while *autonomy* is reflected in the process of obtaining meaningful IC [8]. *Disclosure* pertains to providing the patient with all necessary information to make an informed decision about the procedure [8]. Legally, if a patient signs an IC form without engaging in a dialogue with the physician, the process is deemed invalid and constitutes negligence [9]. Despite the widespread acceptance of IC, a significant gap persists between the theoretical and legal standards of best practice and the way IC is implemented in daily clinical settings [10,11].

Adequate IC dialogues result in additional benefits, such as patient satisfaction, more rapid symptom resolution, reduced emotional distress, lower anxiety, shorter hospitalizations, and lower costs [6,7]. Furthermore, elevated self-efficacy beliefs following an educational dialogue predicted less intense post-operative pain independent of other pre-operative factors [12]. Studies, however, indicate that dialogues were deficient, and the needs of patients often remained unmet [4,5,13,14,15,16,17,18]. Naturally, the time preceding surgery is marked by uncertainty, angst, and anxiety, going beyond health conditions, education, and age [19,20,21]. Studies, however, suggest that the dialogue itself may provoke pre-operative anxiety, and failing to alleviate it may adversely influence the immune system and recovery and cause time delays in surgical treatment [22,23,24]. Previous studies tested the alleviation of pre-operative anxiety.

In one study, 16% of 411 patients felt anxious due to the timing of the disclosure [25]. Another study found that only 21% of patients agreed that discussing the risks that outweighed the benefit provoked anxiety [25]. An overestimation of perioperative mortality risk, common in patients undergoing general surgery, was associated with pre-operative anxiety [22]. Studies that tested the effects on anxiety of various formats of information delivery found that no format improved patient anxiety [26,27]. An exhaustive IC process decreased pre-operative patient anxiety; however, providing the information just before the procedure increased anxiety [26,28]. A study on 3D virtual reality before the procedure improved patients’ comprehension, but failed to decrease anxiety [29]. Also, a virtual reality education intervention resulted in lower post-operative pain scores than those receiving the usual care [30]. Since patients prefer interpersonal communication and not content communication, tailoring pre-operative education to the patient’s style of seeking information reduced pre-operative anxiety, depression, and improved satisfaction with pre-operative education, but the anxiety showed an increase immediately following the pre-operative education [31,32].

A new paradigm views trust of patients in physicians as possibly reducing pre-operative anxiety and restraining medical–legal acts [29]. To reduce anxiety by establishing patient trust, physicians must meet the expectations of patients regarding the IC dialogue so that at the moment of truth, the physician will act in the patient’s best interest [33,34]. If patient experience is short of meeting expectations, distrust is created [35].

IC dialogues aim at promoting the patient’s best interest and may create surgeon–patient trust, but they lack a constructive notion of the surgeon–patient interaction [36]. Although IC is a frequent and integral part of clinical practice, its current doctrine remains mostly a matter of law and ethics, and empirical research to clarify patient expectations is scarce [37]. Therefore, most studies on improving IC processes have sought to enhance patient knowledge rather than to reduce patient anxiety [4]. Previous studies called for further study on how to improve IC processes through improved communication strategies that can minimize risk perception in surgical patients [4,5,6,7,10,11,14,22,38]. This study responds to these calls, seeking to extend the knowledge on alleviation of pre-operative anxiety in the IC process, which, despite its importance, is a less commonly measured outcome. The goals of this study were to (a) map and analyze patient expectations regarding communication in IC, (b) to identify communication that heightens anxiety in the IC process, and (c) to identify communication that reduces anxiety.

## 2. Materials and Methods

This study is part of a research project on expectations of patients throughout the hospitalization phases, from the emergency department to discharge, using the same statistical methods.

### 2.1. Sample

**Figure 1:**
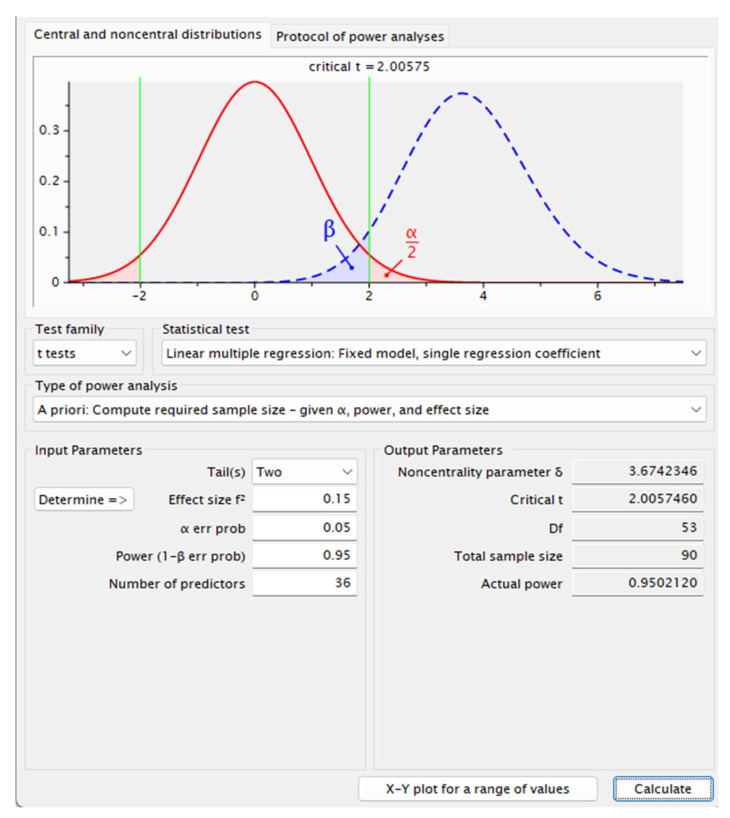
Power analysis results.

A calculation of the sample size with G-Power 3.1 software indicated that 90 subjects would provide a power of 0.95 at a significance level of 0.05 with an effect size of 0.15. Figure 1 presents the power analysis results. Participants were recruited through an online sample provider company, Lucid. Inc. (New Orleans, LA, USA). The inclusion criteria were defined as ages 18 and up who were hospitalized for surgery in the last six months and signed an IC form. The exclusion criteria that were defined were being treated for mental health issues or medication consumption for depression, anxiety, or other mental health issues. The sample size accords with sample sizes of previous studies that tested the stability of coefficients, rather than the stability of means or standard deviations [39,40,41,42].

### 2.2. Design

Since our reality is complex, encompassing many stimuli that may interact with one another, we utilized a conjoint-based experimental design study well acknowledged in both academia and industry for uncovering the power of messages in a great variety of topics [43,44]. Based on Kirk [45], as required in conjoint-based designs, the computerized platform set the design by allocating patients to different groups using repeated measures, where the same patients took part in each condition of each of the independent variables (within groups, or within-subjects design). The computerized conjoint platform alternated the order by which combinations of messages appeared on the screen [46]. Thus, patients rated a series of different combinations of messages, with the same rating question. Patients did not rate “parallel measures”, but were repeatedly exposed to the same question in relation to different aspects of communication with surgeons in the IC dialogue before surgery [46]. This experimental design enabled, compared to typical observational studies or surveys, higher variation, randomization, analysis of co-variance, and control, reducing biases [46]. This conjoint-based experimental design uncovered the power of specific verbal communication messages as affecting patient anxiety in the IC dialogue with surgeons. Numerous messages were tested with no limitation on degrees of freedom [46]. Each patient evaluated a unique set of 48 combinations of verbal messages created by the basic experimental design [46]. With 104 respondents, each rating 48 combinations, this study covered 4992 messages.

### 2.3. Procedure and Materials

Respondents were asked to rate how different combinations of messages regarding verbal communication in IC would increase or decrease their anxiety. The rating question was the same for all respondents, avoiding “repetitions” or “parallel measures”. Messages were obtained by a thorough search of the literature and set into combinations according to a conjoint-based experimental design. Each respondent ranked a set of 48 combinations of messages, each comprising a unique set of messages, independently of each other. The design was set up ahead of time to create specific combinations, with the same structure of the experimental design, for each respondent. The combinations and their order changed by conjoint software (Bimileap, https://www.bimileap.com/). This experimental design sought to uncover the contribution of each statement to the increase or the decrease in anxiety through ordinary least-squares regression (OLS). The rating of the questions ranged from 1 (“decreases anxiety”) to 9 (“increases anxiety”). Respondents were unable to evaluate one statement at a time and were required to assign one number to each combination without much conscious thought. The pattern of ratings for each respondent enabled us to identify which messages, if any, “drove” the ratings. Table 1 presents the literature-based instrument of six categories relating to the IC process and six messages in each.

### 2.4. Data Analysis

The rating questions dealt with the likelihood that a particular combination of verbal communication messages would increase the respondent’s anxiety, leave it unchanged, or decrease the anxiety. Ratings of 1–3 denoted “decreases anxiety”, and ratings of 7–9 denoted “increases anxiety”. To reduce variance, the results were recorded as ratings of 1–6, zero denoting no increase in anxiety, and recoded as ratings of 7–9 to 100, denoting an increase in anxiety. The additive constant for the total panel was 40, meaning that we expected to see about 40% of the combinations receiving a rating of 7–9. The *t*-statistic is the ratio of the coefficient to the standard error, and tells us, through its associated probability, the *p*-value, the likelihood that that real coefficient is 0. We moved directly to the binary transformation, using the original 9-point scale as a preliminary step. Most coefficients in the ordinary least-squares regression for the total panel were not significant. K-means mathematical clustering, based on the similarity in response patterns of respondents to each message regarding whether anxiety would increase or decrease in the IC dialogue yielded three segments. The optimal cluster number was verified using the NbClust R-project package (version 3.0.1) [47].

The following assumption tests for *t*-tests and ordinary least squares regression were carried out: Levene’s test for homogeneity of variances and normality tests using the Kolmogorov–Smirnov test. The data set met all these criteria.

## 3. Results

### Descriptive Analysis

A total of 104 US respondents participated in this explorative online survey on preoperative IC. Female participants were overrepresented in the sample, while the age distributions showed that the panel consisted of older participants (more than 50% were older than 50 years). Most participants identified as Caucasian and more than 50% were married. Almost half of the sample reported a one-year household income ranging between USD 20,000 and USD 49,000 and the majority had obtained an academic degree. About one-third of the respondents reported that they lived in a small household with one or two members (including themselves). The place of residence showed a balanced distribution, as 45% reported that they lived in a suburban area, and 26% and 29% reported city/urban and rural areas, respectively. Table 2 presents sample demographics.

Most participants (62%) reported that they learned about their medical conditions or medical procedures on the internet. About 75% were non-smokers and 61% saw their primary care physician about every 3–6 months. When asked about the number of hospital stays in the past 10 years, 64% reported one to two, 23% reported three to four, 5% reported five, and 8% reported more than six. Of the respondents,72% considered themselves spiritual and 55% did not have a medical power of attorney or living will.

Results of OLS for the entire data set showed significant differences in coefficients only in the case of D2–D6. Table 3 presents these results.

Based on the pattern of coefficients, respondents were divided into groups of individuals with similar response patterns to different verbal messages in the IC dialogue. The number of clusters was verified by NbClust R-package [47]. These groups are mindsets reflecting similar expectations of surgical patients from the IC process. Post hoc ANOVA tests indicated that the differences based on the communication messages among mindsets were significant. Table 4 presents messages by mindset.

Next, we analyzed the messages that respondents rated as messages that would reduce their pre-operative anxiety in verbal communication with physicians in the IC process. To reduce variability, ratings of 1–3 were recoded to 100 to denote reduced anxiety and ratings of 4–9 were recoded to 0 to denote “increases anxiety”. Again, results of ordinary least-squares regression applied to the entire data set showed no significant coefficients. Mathematical k-means clustering was performed [48]. This clustering yielded three distinct mindsets. Post hoc ANOVA tests indicated that the differences based on the communication messages among mindsets were significant. Mindsets are presented in Table 5.

**Figure 2:**
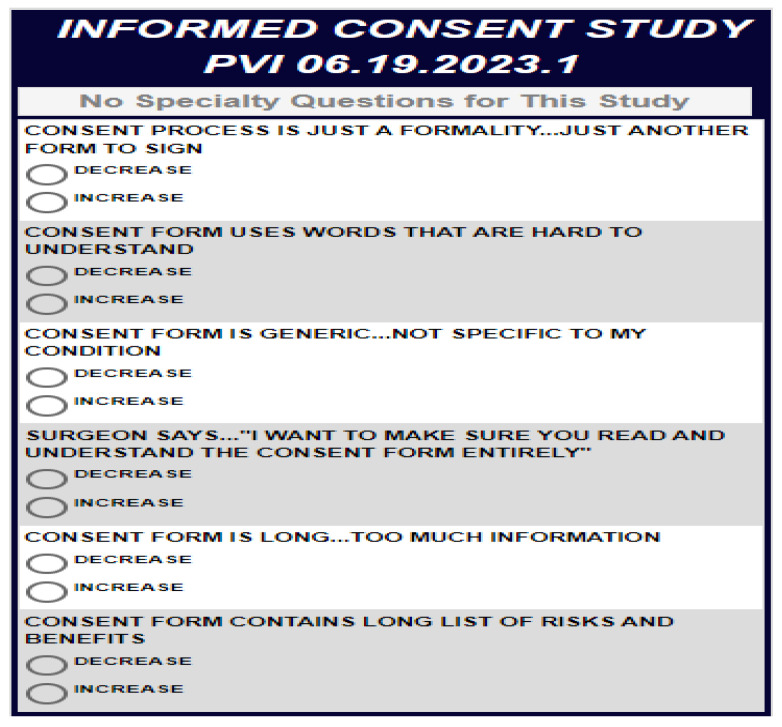
Personal viewpoint identifier created based on the data presented in Table 4. Source: https://www.pvi360.com/TypingToolPage.aspx?projectid=2331&userid=2008 (accessed on 25 February 2025).

Next, to predict the mindset of each respondent, we developed a predictive algorithm. Figure 2 presents the predictive algorithm.

The personal viewpoint identifier algorithm was developed to classify patients into mindsets based on what increases anxiety. PVI took the data presented in Table 4 and looked for the discriminative messages that most differentiated among the three mindsets. These messages were then compared by the respondents, rating binary messages to determine which message increased anxiety more. Using the binary ratings, the PVI calculated the most probable mindset membership of a given respondent. Using the PVI, analysis time decreased significantly, and feedback (e.g., which mindset the given participant belongs to) was presented immediately.

## 4. Discussion

This study mapped and analyzed messages as drivers of pre-operative anxiety in surgical patients regarding verbal communication with surgeons in the pre-operative IC process. This study makes several contributions. Theoretically, this study voices concerns of patients regarding the IC process and extends the knowledge regarding anxiety in the IC process and how to mitigate it. Methodologically, this study utilized a conjoint-based experimental design, overcoming typical biases of surveys. Recommendations based on the insights of this study may support surgeons in choosing what to emphasize in verbal communication with patients based on their mindset to reduce and avoid increasing pre-operative anxiety and enhance well-being and trust in physicians, leading to higher adherence.

The findings suggest that members of the distinct mindsets differ in their expectations and in their drivers of anxiety. These findings stress that different verbal communication messages may increase or decrease anxiety in the IC process depending on mindset. Verbal communication that meets patient expectations in the IC process not only ensures enhanced information provision but also ensures time for a dialogue that targets the emotional needs of patients. The three verbal communication messages that patients thought would decrease their pre-operative anxiety for the whole sample were as follows. Surgeon says, “I want to make sure you read and understand the consent form entirely”. “Everything is provided in clear and simple terms”. Surgeon says, “Let’s go over the entire IC form”. The strongest messages facilitated the name of the mindsets [40,41].

Patients belonging to mindset 1, comprising 70% of the sample, reported that they needed the information to be tailored to their specific condition and situation. These patients thought they would need to feel that everything is taken care of so that all is known and nothing unexpected will happen. They thought they would want to feel that they could “trust” and “understand” what was going on. Patients belonging to mindset 2, comprising 13% of the sample, thought they would need the surgeons themselves to hold a patient-centered dialogue on benefits and risks with them at the clinic, and not at the hospital. They expected the surgeons to have a dialogue with them and would need to hear that the IC process aims at protecting them, not the hospital or the surgeon. They also thought they would like the physician to ask them if they had any other questions or concerns. They thought that what would increase their anxiety was being asked to sign a generic IC form, rather than one specified for their condition. Such a form would make them perceive the IC as protecting the physician and hospital and not protecting them. Last, patients in this mindset thought that the use of words that are hard to understand or medical terminology would increase their anxiety.

Patients belonging to mindset 3, comprising 17% of the sample, experienced high anxiety with all tested verbal communication messages in the IC dialogue, and particularly they thought that lack of information regarding the purpose of signing the IC would cause high anxiety. If they were rushed for time, if they had too little time to thoroughly read the IC form, or if they signed it minutes before the procedure, they would also feel more anxious, as well as if the IC form were too technical and required legal/medical knowledge to understand its content or if they were required to give consent when they were not comfortable regarding the IC. Nothing would drive their anxiety more than not understanding the terminology. This finding supports previous studies on lack of adequate understanding as being particularly common among vulnerable populations who face language barriers or limited health literacy [48].

The findings stress the rationale of the IC process as considering the patient’s unique characteristics, preferences, and feelings when discussing benefits and risks of surgery to avoid anxiety, disputes, and claims and to promote effective recovery [6,7]. The findings also support previous studies on effective IC processes as ones where physicians need to understand patient preferences by discussing risks and benefits of the procedure in question that are particularly relevant for each patient [7]. The findings echo a previous study that stressed that the IC process serves to qualify the patient’s belief system in the physician and the health system and to establish patient trust [49]. Only then is the physician able to support the patient in weighing possible benefits and harms and making a decision with significant autonomy.

Unresolved doubts of patients and fears may inhibit patient trust in both the physician and the IC process. The IC dialogue can help set aside fears of patients regarding complications and outcomes, leading to the patients feeling that the physician understands and respects their concerns and is actively listening to them [50,51]. Insights of this study call upon physicians to reduce anxiety by focusing attention on the needs of each patient rather than on the constraints of the hospital and urge physicians to keep patients informed, improve communication with patients, support patients, elicit feedback from them, listen to patients’ views, and be open and candid [6,7,52]. Improvement in IC processes needs support from physicians, as it plays a major role in forming a therapeutic alliance with the patient [53]. Moreover, experience and learning non-technical attributes, such as communication with patients, develop over time [54,55]. Since physicians are responsible for the consent obtained in the IC dialogue, they may also take responsibility for improving the verbal communication in the IC dialogue by using the predictive algorithm that we developed to ensure that the discussion includes verbal communication that is tailored to the needs of patients as individuals and decrease their anxiety [10].

The novel strategy we demonstrated in this study on expectations of patients from dialogues with surgeons in the IC process is not without limitations. First, while the presented statistical strategy itself is replicable and generalizable across health settings and countries, mindsets of patients are rooted in culture, reducing the generalizability of the study. Second, there are differences in attitudes towards surgeons that may affect the expectations of patients from surgeons in the IC dialogue. Third, we did not test patient expectations by medical specialty, which may yield different mindsets resulting in varying importance levels of the variegated messages for each mindset. Future studies may test the expectations of patients before surgery by medical specialty and culture. Also, future studies may test the effect of implementing mindset-tailored verbal messages in IC dialogues.

## 5. Conclusions

Training to improve IC should consider not only how to deliver information but also how to structure the IC dialogue to reduce patient anxiety by targeting expectations and needs of patients and obtaining the skills for discussing plans and for shared decision-making of patients. IC is most likely to be achieved when the patient has had a dialogue with a physician who is not only skilled at enhancing the patient’s knowledge but also willing to provide patients with the opportunity to deliberate on the choices available to them and then to be able to express their decision without feeling anxious or pressured. Physicians are not specifically trained on carrying out a dialogue in the IC process and lack the competence to guide patients through a legally correct IC process, which is an integral part of surgical practice. The predictive algorithm enables physicians to quickly identify the belonging of each patient at the clinic to a sample/mindset, facilitating patient-tailored communication by mindset.

## Figures and Tables

**Table 1:** Instrument designed by a conjoint-based experimental design.

	**Question A—What is “Unfriendly” about the IC?**
A1	The informed consent form (ICF) is long… too much information.
A2	The ICF uses words that are hard to understand.
A3	The ICF is specific to my condition.
A4	The ICF contains a long list of risks and benefits.
A5	The ICF is generic… not specific to my condition.
A6	The ICF is too technical, I need legal/medical knowledge to understand.
	**Question B—How can the consent form be friendly?**
B1	The IC process is patient-centered, educating on risks/benefits of procedure.
B2	The IC process only for signing form… just protects hospital and doctor.
B3	The IC process… full discussion of the risks/benefits/alternatives.
B4	Giving consent is more than just a legal release.
B5	The consent process is there to protect you… the patient.
B6	The consent process is just a formality… just another form to sign.
	**Question C—What IC process makes you feel comfortable?**
C1	Receive information both orally and written… patient’s fundamental right.
C2	Everything is provided in clear and simple terms.
C3	Information is tailored to me… to my situation.
C4	Afterwards… I can explain back important information… understand the conversation.
C5	A fact sheet is given to explain why IC is important.
C6	Once informed, you could decline the procedure.
	**Question D—What is a situation in which the IC process fails?**
D1	Requires giving consent when you’re not feeling well.
D2	I don’t understand the medical terminology.
D3	I’m embarrassed to reveal to the medical staff that I don’t understand.
D4	I’m rushed and there is not enough time to make a decision
D5	There is too little time given to read the consent form.
D6	Lack of information… What am I signing? What’s the purpose?
	**Question E—What is the language you prefer that the surgeon to use?**
E1	Surgeon says, “This form gives me permission to perform the procedure… sign here.”
E2	Surgeon says, “Do you have any other questions or concerns?”
E3	Surgeon says, “Let’s go over the entire consent form.”
E4	Surgeon says, “Let me highlight only relevant information.”
E5	Surgeon says, “I want to make sure you read and understand the consent form entirely.”
E6	The surgeon is the one explaining things.
	**Question F—How may the IC process occur?**
F1	Sign consent form minutes before procedure.
F2	Receive the form to read days prior to procedure… when possible.
F3	Give consent over the phone.
F4	Get the IC done in the doctor’s office.
F5	Obtain IC over electronic means… video call, FaceTime, Skype.
F6	Give consent when family is present for better decision-making.

**Table 2:** Demographic characteristics of the sample.

Demographic Variable	Levels	N	Percentage
Sex	Male	37	36%
	Female	67	64%
Age (years)	18–29	3	3%
	30–39	23	22%
	40–49	13	12%
	50–59	23	22%
	60–69	19	18%
	70–79	20	19%
	80 years and older	3	3%
Race	White	85	82%
	Black/African American	12	12%
	Hispanic	2	2%
	Native American	1	1%
	Asian	2	2%
	Other	2	2%
Marital status	Married	53	51%
	Divorced	18	17%
	Never married	22	21%
	Widow/widower	11	11%
Household income in the last 12 months, USD	<20,000	19	18%
	20,000 to 49,000	47	45%
	50,000 to 99,000	28	27%
	100,000 to 149,000	7	7%
	150,000 to 199,000	3	3%
Level of education	Less than high school	0	0%
	High school or equivalent	25	24%
	Some college	27	26%
	Associate degree	18	17%
	Bachelor’s degree	24	23%
	Graduate or professional degree	10	10%
Size of the household (including the respondent)	1	26	25%
	2	42	40%
	3	14	13%
	4	17	16%
	5	2	2%
	6 or more	3	3%
Place of residence	City/urban	27	26%
	Suburban	47	45%
	Rural	30	29%

**Table 3:** Anxiety-increasing verbal communication messages for IC for the total sample *.

	IC—Increased Anxiety	Value	S.E.
	Additive Constant	40.3	
A1	The ICF is long, too much information.	2	0.03
A2	The ICF uses words that are hard to understand.	6	0.19
A3	The ICF is specific to my condition.	−3	0.09
A4	The ICF contains long list of risks and benefits.	−1	0.08
A5	The ICF is generic… not specific to my condition.	4	0.12
A6	The ICF is too technical… need legal/medical knowledge to understand.	2	0.05
B1	The IC process is patient centered educating on risks/benefits of procedure.	−6	0.17
B2	The IC process for signing form… just protects hospital and doctor.	1	0.03
B3	The IC process… full discussion of the risks/benefits/alternatives.	−4	0.26
B4	Giving consent is more than just a legal release.	−1	0.06
B5	The IC process is there to protect you… the patient.	−3	0.05
B6	The IC process is just a formality… just another form to sign.	−2	0.07
C1	Receive information both orally and written… patient’s fundamental right.	−5	0.49
C2	Everything is provided in clear and simple terms.	−10	0.25
C3	Information is tailored to me… my situation.	−7	0.26
	Afterwards, I can explain back important information, understand the conversation.	−1	0.05
C5	A fact sheet given to explain why informed consent is important.	−6	0.05
C6	Once informed, you could decline the procedure.	−3	0.04
D1	Requiring giving consent when I’m not feeling well.	6	0.04
D2	Don’t understand the medical terminology.	**8**	0.73
D3	Embarrassed to reveal to the medical staff that I don’t understand.	**9**	0.81
D4	IC is rushed, not enough time to make a decision	**12**	1.11
D5	Too little time given to actually read the consent form	**11**	0.29
D6	Lack of information… What am I signing? What’s the purpose?	7	0.32
E1	Surgeon says, “This form gives me permission to perform the procedure… sign here”	2	0.13
E2	Surgeon says, “Do you have any other questions or concerns?”	−8	0.59
E3	Surgeon says, “Let’s go over the entire consent form”	−12	0.58
E4	Surgeon says, “Let me highlight only relevant information”	−1	0.02
E5	Surgeon says, “I want to make sure you read and understand the consent form entirely”	−10	0.05
E6	The surgeon is the one explaining things	−6	0.23
F1	Sign consent form minutes before procedure	7	0.02
F2	Receive the form to read days prior to procedure when possible	−7	0.4
F3	Ok to give consent over the phone	3	0.17
F4	Get it done in the doctor’s office	0	0.01
F5	Obtain over electronic means… video call, FaceTime, Skype	0	0.03
F6	Consent given when family present… better decision-making	−6	0.46

* Coefficients of +8 or higher are significant (<0.001).

**Table 4:** Anxiety-increasing verbal communication messages for IC by mindset (MS) and ANOVA post-hoc tests *.

	IC—Increased Anxiety	MS 1	MS2	MS 3
		**63%**	**18%**	**19%**
	Additive Constant	35b	73a	153c
A1	The ICF is long, too much information	5b	58c	−34a
A2	The ICF uses words that are hard to understand	6b	**49c**	−33a
A3	The ICF is specific to my condition	2b	**37c**	−41a
A4	The ICF contains long list of risks and benefits	1b	**43c**	−46a
A5	The ICF is generic… not specific to my condition	**12b**	**43c**	−58a
A6	The ICF is too technical, I need legal/medical knowledge to understand	0b	**44c**	−28a
B1	The ICF is patient-centered, educating about risks/benefits of procedure	−4b	**32c**	−39a
B2	The IC process only for signing form… just protects hospital and doctor.	−1b	**33c**	−25a
B3	The IC process… full discussion of the risks/benefits/alternatives	−1b	**35c**	−37a
B4	Giving consent is more than just a legal release	1b	**47c**	−22a
B5	The IC process is there to protect you… the patient	−2b	**45c**	−27a
B6	The IC process is just a formality… just another form to sign	−1a	**51b**	−19a
C1	Receive information both orally and written… patient’s fundamental right	−2b	**36c**	−29a
C2	Everything is provided in clear and simple terms	−0b	**17b**	−30a
C3	Information is tailored to me… my situation	−6b	**40c**	−33a
	Afterwards… I can explain back important information… understand the conversation	0a	**34b**	−21a
C5	A fact sheet is given to explain why informed consent is important	−4b	**34c**	−40a
C6	Once informed, you could decline the procedure	0a	**23b**	−19a
D1	Requiring giving consent when I’m not feeling well	**8b**	**47c**	−23a
D2	Don’t understand the medical terminology	**8b**	**39c**	−13a
D3	Embarrassed to reveal to the medical staff that I don’t understand	5b	**42c**	−27a
D4	IC is rushed, not enough time to make a decision	**10b**	**46c**	−20a
D5	Too little time is given to actually read the consent form	**13b**	**32b**	0a
D6	Lack of information… What am I signing? What’s the purpose?	6b	**44c**	−22a
E1	Surgeon says, “This form gives me permission to perform the procedure… sign here”	−2a	**22b**	−25a
E2	Surgeon says, “Do you have any other questions or concerns?”	−5b	2b	−34a
E3	Surgeon says, “Let’s go over the entire consent form”	−18a	**17b**	−44a
E4	Surgeon says, “Let me highlight only relevant information”	0b	**25c**	−36a
E5	Surgeon says, ”I want to make sure you read and understand the consent form entirely”	−3.7b	**10b**	−51a
E6	The surgeon is the one explaining things	−6b	**26c**	−37a
F1	Sign ICF minutes before procedure	3b	**47c**	−33a
F2	Receive the ICF to read days prior to procedure… when possible	−4b	**18b**	−38a
F3	Ok to give consent over the phone	−6b	**18b**	−29a
F4	Get it done in the doctor’s office	−1b	**19b**	−36a
F5	Obtain over electronic means… video call, FaceTime, Skype	−0b	**20b**	−39a
F6	Consent given when family present… better decision-making	−15b	**8c**	−45a

* Coefficients of +8 or higher are significant (<0.001).

**Table 5:** The anxiety-decreasing power of messages for IC by coefficient of distinct mindsets (MS) and ANOVA post hoc indices * (n = 104, additive constant = 16).

	IC—Decreased Anxiety	MS 1	MS 2	MS 3
	SIZE	**73**	**13**	**18**
	Additive Constant	11b	104a	123c
A1	The ICF is long, too much information	−5b	48c	−36a
A2	The ICF uses words that are hard to understand	−5b	32c	−30a
A3	The ICF is specific to my condition	4b	44c	−16a
A4	The ICF contains long list of risks and benefits	−1b	44c	−30a
A5	The ICF is generic… not specific to my condition	−3b	**48c**	−42a
A6	The ICF is too technical, I need legal/medical knowledge to understand	−3b	24c	**−49a**
B1	The IC process is patient-centered, educating on risks/benefits of procedure	5a	**51b**	−7a
B2	IC process only for signing form, just protects hospital and doctor	1b	**48c**	−18a
B3	IC process… full discussion of the risks/benefits/alternatives	6b	26c	−13a
B4	Giving IC is more than just a legal release	5b	35c	−14a
B5	The IC process is there to protect you… the patient	6b	40c	−17a
B6	The IC process is just a formality… just another form to sign	−2a	22b	−20a
C1	Receive information both orally and written about your fundamental rights	6b	25b	−19a
C2	Everything is provided in clear and simple terms	2b	**47c**	−26a
C3	Information is tailored to me and my situation	**9b**	44c	−19a
C4	Afterwards, I can explain back important information, understand the conversation	−2b	26c	−33a
C5	A fact sheet is given to explain why IC is important	7b	37c	−31a
C6	Informed, you have the opportunity to decline the procedure	1b	42c	−37a
D1	Requiring giving consent when I’m not feeling well	0b	8b	**−49a**
D2	Don’t understand the medical terminology	−5b	20c	−26a
D3	Embarrassed to reveal to the medical staff that I don’t understand	−1b	19c	−32a
D4	IC is rushed, not enough time to make a decision	−7b	13b	**−50a**
D5	Too little time given to actually read the consent form	2.b	13b	−39a
D6	Lack of information on What am I signing? What’s the purpose?	−5b	27c	**−45a**
E1	Surgeon says, “This form gives me permission to perform the procedure… sign here”	1a	39b	−16a
E2	Surgeon says, “Do you have any other questions or concerns?”	3a	**55b**	−2a
E3	Surgeon says, “Let’s go over the entire consent form”	**9a**	39b	−7a
E4	Surgeon says, “Let me highlight only relevant information”	−2b	38c	−31a
E5	Surgeon says, “I want to make sure you read and understand the ICF entirely”	**8b**	38c	−13a
E6	The surgeon is the one explaining things	4b	**46c**	−15a
F1	ICF is signed minutes before procedure	−4b	34c	−40a
F2	Receive the form to read days prior to procedure, when possible	4b	31c	−17a
F3	Ok to give consent over the phone	2b	32c	−18a
F4	Get it done in the doctor’s office	1b	**48c**	−18a
F5	Obtain over electronic means like video call, FaceTime, Skype	−4b	39c	−29a
F6	IC is given when the family is present, better decision-making.	7b	43c	−25a

* Coefficients of +7.51 or higher are significant (*p* < 0.001).

## Data Availability

The raw data supporting the conclusions of this article will be made available by the authors on request.
